# Patients' and doctors’ views and experiences of the patient safety trajectory of breast cancer care

**DOI:** 10.1016/j.breast.2024.103699

**Published:** 2024-02-29

**Authors:** Clara Forrest, Martin J. O'Sullivan, Max Ryan, Colm O'Tuathaigh, Tara Jane Browne, Kathy Rock, Mary Jane O'Leary, Deirdre Madden, Seamus O'Reilly

**Affiliations:** a Academic Track Intern Programme, Intern Network Executive, School of Medicine, University College Cork, Cork, Ireland; b Department of Surgery, Cork University Hospital, Wilton, Cork, Ireland; c Cancer Research@UCC, College of Medicine and Health, University College Cork, Cork, Ireland; d Department of Radiology, Cork University Hospital, Wilton, Cork, Ireland; e Medical Education Unit, School of Medicine, University College Cork, Cork, Ireland; f Department of Histopathology, Cork University Hospital, Wilton, Cork, Ireland; g Department of Radiation Oncology, Cork University Hospital, Wilton, Cork, Ireland; h Department of Palliative Medicine, Marymount University Hospice and Hospital, Bishopstown, Cork, Ireland; i School of Law, University College Cork, Cork, Ireland; j Department of Medical Oncology, Cork University Hospital, Wilton, Cork, Ireland

**Keywords:** Patient safety, Medical error, Breast cancer, Litigation, Medical negligence

## Abstract

**Introduction:**

Successful breast cancer outcomes can be jeopardised by adverse events. Understanding and integrating patients' and doctors’ perspectives into care trajectories could improve patient safety. This study assessed their views on, and experiences of, medical error and patient safety.

**Methods:**

A cross-sectional, quantitative 20–40 item questionnaire for patients attending Cork University Hospital Cancer Centre and breast cancer doctors in the Republic of Ireland was developed. Domains included demographics, medical error experience, patient safety opinions and concerns.

**Results:**

184 patients and 116 doctors completed the survey. Of the doctors, 41.4% felt patient safety had deteriorated over the previous five years and 54.3% felt patient safety measures were inadequate compared to 13.0% and 27.7% of patients respectively. Of the 30 patients who experienced medical errors/negligence claims, 18 reported permanent or long-term physical and emotional effects. Forty-two of 48 (87.5%) doctors who experienced medical errors/negligence claims reported emotional health impacts. Almost half of doctors involved in negligence claims considered early retirement. Forty-four patients and 154 doctors didn't experience errors but reported their patient safety concerns. Doctors were more concerned about communication and administrative errors, staffing and organisational factors compared to patients. Multiple barriers to error reporting were highlighted.

**Conclusion:**

This is the first study to assess patients' and doctors’ patient safety views and medical error/negligence claims experiences in breast cancer care in Ireland. Experience of medical error/negligence claims had long-lasting implications for both groups. Doctors were concerned about a multitude of errors and causative factors. Failure to embed these findings is a missed opportunity to improve safety.

## Highlights

• Many doctors have negative patient safety views and are more negative than patients.
• Medical errors have long-term physical, emotional and financial impacts on patients.
• Experience of negligence claims impacts doctors' choices about early retirement.
• Multiple barriers exist to reporting errors or concerns for patients and doctors.

## 1 Introduction

Breast cancer is the most common malignancy involved in malpractice claims in America [1]. At diagnosis, evidence-based guidelines outline the stages of patients’ breast cancer diagnostic and therapeutic trajectories [2]. Both patients and doctors may not envisage an additional trajectory: one of medical error which runs parallel to each of these diagnostic and therapeutic interventions, and whose components relate to safety, risk and error.

The World Health Organisation (WHO) define patient safety as “the prevention of errors and adverse effects to patients associated with healthcare” [3]. Medical errors, defined as “an adverse event or near miss that is preventable with the current state of medical knowledge”, are common and many are preventable [[3], [4], [5], [6]]. Patient safety could be considered the twenty-first-century version of *‘primum non nocere*’ (‘first do not harm’). However, abiding by this principle is increasingly difficult because knowledge is expanding faster than our ability to apply it. The doubling time of medical knowledge increased from 50 years in 1950 to 73 days in 2020 [7]. Expanding treatment options have led to growing complexity of care and increasing risk as “every point in the process of care can contain an inherent risk” [8].

Pivotal studies have identified that hundreds of thousands of deaths annually were the result of inadequate safety practices and medical errors [9,10]. Adverse events also have objective financial consequences [4,6]. When a medical negligence lawsuit follows an error, the cumulative costs can jeopardise the financial sustainability of public cancer screening programmes or even entire public healthcare systems [[11], [12], [13]]. Patient safety improvement is key to avoiding the consequences of medical error, and stakeholder involvement is central to this [8]. However, there is scant research using stakeholder involvement to examine the oncological patient safety trajectory.

The present study aimed to interrogate the patient safety dimensions of the breast cancer trajectory through a quantitatively-based, multi-stakeholder approach. Engagement of patients has been described by the WHO as “the most powerful tool to improve patient safety” [8]. Prior studies show they hold contrasting views and identify different event types, contributing factors and outcomes compared to doctors [14,15]. We therefore solicited the views of both breast cancer patients and treating doctors to assess their insights into the patient safety trajectory. Secondly, the experiences of those who were involved in a medical error, as well as the concerns of those who had not, were solicited to identify perspectives on how patient safety could be improved.

## 2 Methods and materials

A cross-sectional, quantitative questionnaire study assessing the patient safety views and experiences of breast cancer patients, and doctors providing breast cancer care was conducted. Questionnaire design was informed by three published sources on patient safety and mitigating medical error and had 20-40 questions depending on the respondents' preceding answers [[16], [17], [18], [19]]. The WHO's definitions of patient safety, medical error, adverse event and near-miss were outlined at the beginning [3]. Questionnaire domains included demographics, patient safety opinions, medical error experience and patient safety concerns. Items were a mixture of binary and non-binary categorical items, as well as Likert scale items. Close-ended questions afforded anonymity.

Patients with breast cancer who had completed or were currently undergoing treatment at the Cork University Hospital Cancer Centre and doctors working nationally with breast cancer care comprised the study population. Between April–June 2022, non-probability sampling was utilised for both groups. The Clinical Research Ethics Committee of the Cork Teaching Hospitals provided ethical approval. Categorical variables were analysed using descriptive statistics. Pearson Chi-square analyses and Fisher's Exact test were used to examine associations between categorical variables [20,21]. Z-test analysis and Bonferroni method of adjustment of *p*-value were utilised for pairwise comparisons of proportions and to correct for Type 1 error respectively [22,23].

## 3 Results

### 3.1 Patients' and doctors' views on patient safety and medical error

In total, 184 patients and 116 doctors responded to the survey between April–June 2022 (Table 1). Most patients (116/184; 64.1%) were first diagnosed within the previous four years. Two-thirds of doctors (77/116; 66.4%) had over 10 years’ experience.

**Fig. 1:**
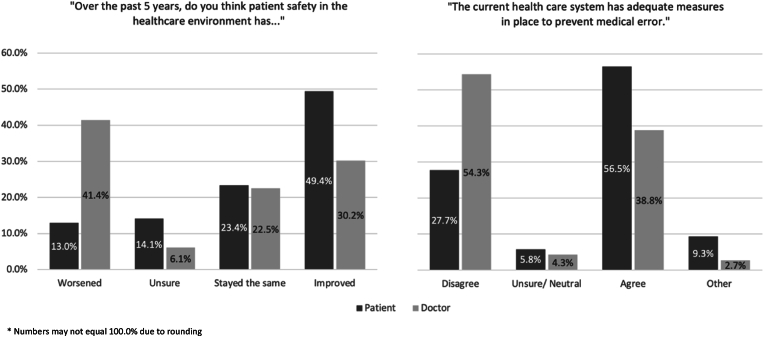
Patients' and doctors' views on patient safety and medical error prevention. * Numbers may not equal 100.0% due to rounding.

Overall, doctors had an unfavourable view of patient safety and medical error prevention compared to patients (Fig. 1). Three times the proportion of doctors (48/116; 41.4%) felt patient safety had worsened in the past five years compared to patients (24/184; 13.0%) (*p* < 0.001). A greater percentage of patients (92/184; 49.4%) felt it had improved compared to doctors (35/116; 30.2%) (*p* < 0.001). After having the terms ‘medical error’, ‘adverse event and ‘near-miss’ defined, 30 patients and 65 doctors reported involvement in a medical error (Table 1). However, when the responses of those who had, and had not, experienced a medical error were compared, there was no significant difference for patients (*p* = 0.157) or doctors (*p* = 0.875).

**Table 1:** Demographics of respondents.

*Demographics of patients*		*Demographics of doctors*	
Number	184	Number	116
Gender (%)		Gender (%)	
Female	180 (97.8)	Female	72 (62.1)
Male	4 (2.2)	Male	42 (36.2)
Age groups (%)		Prefer not to say	2 (1.7)
18–34	5 (2.7)	Age groups (%)	
35–39	10 (5.4)	25–34	20 (17.3)
40–44	22 (12.0)	35–39	9 (7.8)
45–49	19 (10.3)	40–44	23 (19.8)
50–54	25 (13.6)	45–49	25 (21.6)
55–59	37 (20.1)	50–54	15 (12.9)
60+	66 (35.9)	55–59	10 (8.6)
Patient or family member work in healthcare (%)		60+	14 (12.1)
Yes	42 (22.8)	Job title (%)	
No	142 (77.1)	Consultant	92 (79.3)
Residence (%)		Senior/specialist registrar	21 (18.1)
Rural area	60 (32.6)	Junior registrar	3 (2.6)
Small town	53 (28.8)	Field of medicine	
Suburb	38 (20.7)	Medical oncology	34 (29.3)
City	31 (16.8)	Radiation oncology	28 (24.1)
Other	2 (1.1)	Radiology	25 (21.6)
Currently have private health insurance (%)		Surgery	20 (17.2)
Yes	103 (56.0)	Palliative medicine	5 (4.3)
No	78 (42.2)	Pathology	4 (3.4)
Other	3 (1.6)	Experience with breast cancer patients (%)	
First diagnosed with breast cancer (%)		<1 year	4 (3.4)
<1 year	36 (19.6)	1–2 years	6 (5.2)
1–2 years	31 (16.8)	3–5 years	10 (8.6)
3–4 years	51 (27.7)	5–10 years	19 (16.4)
5–6 years	17 (9.2)	10–20 years	42 (36.2)
7–8 years	8 (4.3)	>20 years	35 (30.2)
9–10 years	7 (3.8)	Practice in a private capacity (%)	
>10 years	33 (17.9)	Yes	53 (45.7)
Unsure	1 (0.5)	No	63 (54.3)
Perception of medical error involvement (%)		Perception of medical error involvement (%)	
Yes	30 (16.3)	Yes	65 (56.0)
No	154 (83.7)	No	51 (44.0)
Breast cancer type (%)			
Hormone sensitive invasive ductal carcinoma	39 (21.2)		
Ductal carcinoma in-situ	11 (6.0)		
HER2 positive carcinoma	29 (15.8)		
Triple negative carcinoma	16 (8.7)		
Invasive lobular carcinoma	7 (3.8)		
Metastatic carcinoma	29 (15.8)		
Unsure	46 (25.0)		
Other	7 (3.8)		

The proportion of doctors (63/116; 54.3%) who disagreed that there were adequate medical prevention measures in place was higher than the proportion of patients (51/184; 27.2%) (*p* < 0.001). A higher percentage of patients agreed with the statement (104/184; 56.5%) compared to doctors (45/116; 38.3%) (*p* < 0.001). Prior experience of a medical error had no impact on patients' (*p* = 0.121) or doctors’ responses (*p* = 0.537).

**Fig. 2:**
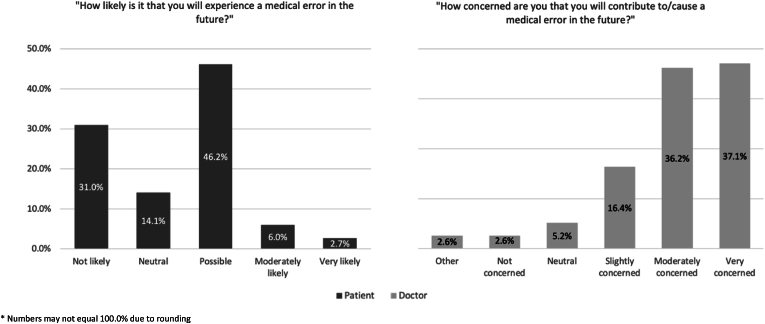
Patients' and doctors' views on future medical error. * Numbers may not equal 100.0% due to rounding.

Fig. 2 demonstrates similar sentiments for the future. Three-quarters of doctors (85/116; 73.3%) were moderately or very concerned about contributing to or causing a future medical error. In contrast, few patients (13/184; 8.7%) felt it was moderately or very likely they would experience a future medical error. Responses did not differ significantly based on previous medical error experience for patients (*p* = 0.057) or doctors (*p* = 0.883).

### 3.2 Patients' and doctors' experiences of medical error during breast cancer care

Of the 300 respondents, 30 patients and 48 doctors expanded on their involvement in a medical error or negligence claim during breast cancer care. Table 1S (supplementary material) displays the 78 respondents’ demographics.

Of the patients who experienced a medical error only, 92.3% (24/26) reported an impact on emotional health, 80.8% (21/26) on physical health and 46.2% (12/26) on financial health (Table 2). The proportion of patients who reported a permanent physical effect (8/26; 30.8%) was higher than doctors (1/35; 2.9%) (*p* < 0.001). Forty-two of 48 doctors (87.5%) reported emotional health impacts. However, this was short-term for a greater percentage of doctors (23/35; 65.7%) than patients (8/26; 30.8%) (*p* = 0.004).

**Table 2:** Physical, emotional and financial impact of experience on patients and doctors.

	Medical error			Medical negligence claim			Total
	*Patient*	*Doctor*	*Total*	*Patient*	*Doctor*	*Total*	
Number	26	35	*61*	4	13	*17*	*78*
Physical health (%)							
Permanent effect	8 (30.8) _a_	1 (2.9) _b_	*9 (14.8)*	3 (75.0) _a_	1 (7.7) _b_	*4 (23.5)*	*13 (16.7)*
Long-term effect	7 (26.9)	2 (5.7)	*9 (14.8)*	0	0	*0*	*9 (11.5)*
Short-term effect	6 (23.1)	12 (34.3)	*18 (29.5)*	0	5 (38.5)	*5 (29.4)*	*23 (29.5)*
No effect	5 (19.2) _a_	20 (57.1) _b_	*25 (40.9)*	1 (25.0)	7 (53.8)	*8 (47.0)*	*33 (42.3)*
Emotional health (%)							
Permanent effect	7 (26.9)	2 (5.7)	*9 (14.8)*	3 (75.0)	2 (15.4)	*5 (29.4)*	*14 (17.9)*
Long-term effect	9 (34.6)	6 (17.1)	*15 (24.5)*	0	5 (38.5)	*5 (29.4)*	*20 (25.6)*
Short-term effect	8 (30.8) _a_	23 (65.7) _b_	*31 (50.8)*	0	4 (30.8)	*4 (23.5)*	*35 (44.9)*
No effect	2 (7.7)	4 (11.4)	*6 (9.8)*	1 (25.0)	2 (15.4)	*3 (17.6)*	*9 (11.5)*
Finances (%)							
Permanent effect	3 (11.5)	1 (2.9)	*4 (6.5)*	1 (25.0)	0	*1 (5.8)*	*5 (6.4)*
Long-term effect	8 (30.8) _a_	0 _b_	*8 (13.1)*	1 (25.0)	0	*1 (5.8)*	*9 (11.5)*
Short-term effect	1 (3.8)	0	*1 (1.6)*	1 (25.0)	0	*1 (5.8)*	*2 (2.6)*
No effect	14 (53.8) _a_	34 (97.1) _b_	*48 (78.7)*	1 (25.0) _a_	13 (100) _b_	*14 (82.4)*	*62 (79.5)*

Each subscript letter denotes a subset of the variable whose column proportions differ significantly from each other at the 0.05 level.

Table 3 outlines the impact of doctors’ experiences on career choices. Overall, one in four doctors (12/48; 25.0%) have considered early retirement. This increases to almost half (6/13; 46.2%) when examining only those involved in a medical negligence claim.

**Table 3:** Impacts of experience on doctors’ career choices.

	Medical error		Medical negligence claim		Total
Number	35		13		*48*
Experience prompted consideration of early retirement (%)					
Yes	6 (17.1)		6 (46.2)		*12 (25.0)*
No	27 (77.1)		6 (46.2)		*33 (68.8)*
Other	2 (5.7)		1 (7.7)		*3 (6.3)*
Experience prompted consideration of working abroad (%)					
Yes	6 (17.1)		2 (15.4)		*8 (16.7)*
No	29 (82.9)		11 (84.6)		*40 (83.3)*
Experience prompted consideration of career change (%)					
Yes	3 (8.6)		2 (15.4)		*5 (10.4)*
No	32 (91.4)		11 (84.6)		*43 (89.6)*

**Fig. 3:**
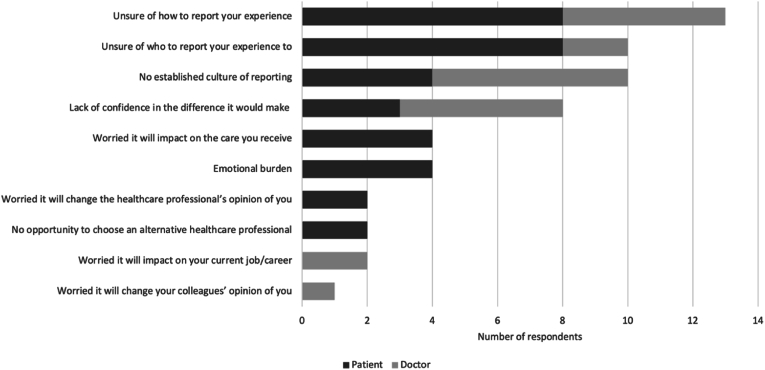
Barriers to reporting medical error.

Of the 61 respondents who experienced a medical error only, the error was reported in 37 instances. The most commonly cited outcome of reporting by doctors was ‘improved communication between healthcare professionals ‘(n = 9). However, for patients, it was ‘improved communication with them’ (n = 4). Fig. 3 shows the most commonly cited barriers to reporting medical errors.

### 3.3 Patients' and doctors' patient safety concerns during breast cancer care

Of the 300 respondents, 154 patients and 44 doctors with no involvement in a medical error reported their patient safety concerns during breast cancer care. Table 2S (supplementary material) displays their demographics.

**Fig. 4:**
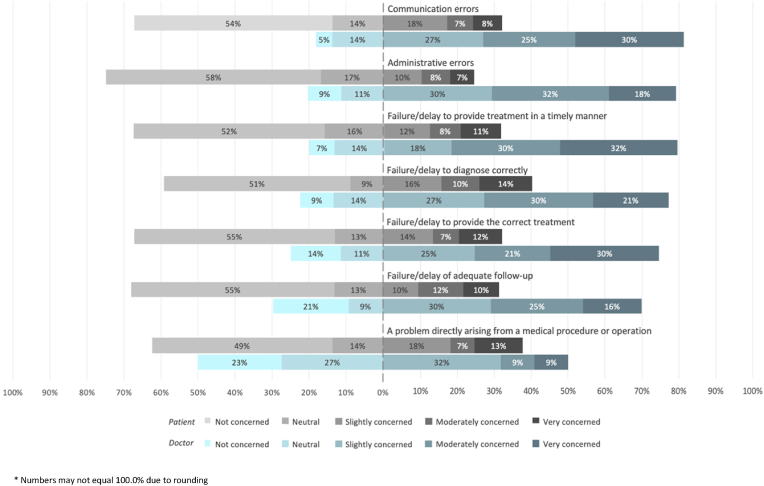
Patients' and doctors' levels of concern for adverse events. * Numbers may not equal 100.0% due to rounding.

Overall, a greater proportion of doctors reported concern for all adverse event types (Fig. 4) (*p* < 0.05*)*. ‘Communication errors’ ranked the highest for doctors with 81.8% (36/44) reporting some concern compared to 32.4% of patients (50/154). The proportion of doctors (22/44; 50.0%) who were moderately or very concerned about ‘administrative errors’ was three times that of patients (22/154; 14.3%) (*p* = 0.001). The largest proportion of patients were concerned about ‘failure or delay to diagnose correctly’ (62/154; 40.3%).

A greater proportion of doctors were concerned about all causative or contributory factors of medical compared to patients (*p* < 0.001). More doctors reported concern about workforce factors such as ‘staff contending with a very high workload’ (40/44; 90%), ‘overworked, burnt-out or stressed staff’ (39/44; 88.6%) and ‘inadequate staffing’ (37/44; 84.1%) compared to patients (60/154; 39.0%, 55/154; 35.7%, 31/154; 20.1% respectively) (*p* < 0.001). Seventy percent of doctors (31/44) were concerned about ‘poorly organised systems for record-keeping’ compared to 24.0% of patients (37/154). ‘Poorly organised systems for appointments and follow-up’ concerned 61.4% of doctors (27/44) compared to just 7.8% of patients (12/154).

**Fig. 5:**
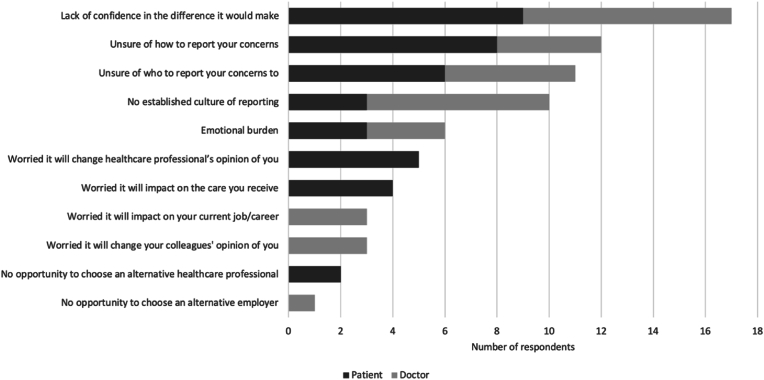
Barriers to reporting concerns.

Of the 198 respondents, 13 patients and 15 doctors reported their patient safety concerns. For patients, the most commonly cited outcome was ‘improved communication between healthcare professionals’ (n = 6). In contrast, for doctors, it was ‘nothing’ (n = 9). For those who did not report their concerns, the most frequent barrier was ‘a lack of confidence in the difference it would make’ (Fig. 5).

## 4 Discussion

Doctors in this study had a more pessimistic outlook on patient safety compared to patients. The majority felt patient safety had worsened or stayed the same in the past five years and that there were inadequate medical error prevention measures. Negative patient safety views are commonplace amongst doctors including in cancer care [24,25]. However, patients in the present study reported more positive attitudes overall regarding risk of medical error than those in larger American studies [18,19]. Most doctors in this study had worked for over a decade in breast cancer care and their concerns should serve as a warning for responsible policymakers. For instance, no reference is made specifically to patient safety in the European Commission's recent ‘Europe's Beating Cancer Plan’ [26]. If not prioritised by policymakers and professional societies, the concept of ‘safety mindfulness’ proposed by Chera and colleagues which is "a recognition that unforeseen errors will occur, and a desire to proactively improve their systems and processes” will not succeed [25].

While landmark publications such as ‘To Err Is Human’ have increased medical error awareness, excessive fear of error can lead to negative defensive practices such as unnecessary investigation, prescribing and hospitalisation which have implications for finite healthcare resources [9,27]. Almost 90% of doctors in this study were concerned about contributing to or causing a future medical error, irrespective of previous experience. A greater proportion of doctors were concerned about various adverse events and causative factors compared to patients. Shifting focus away from fearing medical error and towards improving patient safety is needed to reduce resource-consuming defensive practices [28].

However, such improvement is dependent on data and reporting. Doctors are significantly less likely to report events compared to other healthcare professionals [29]. In total, 37 of the 61 medical errors in this study were reported which is in keeping with an estimated incident reporting rate of 50% in Ireland [6]. As shown in this study, uncertainty surrounding the reporting process reduced compliance by patients and doctors. An active outreach to patients undergoing cancer treatment has been found to identify system deficits in real-time and implement change [30].

For doctors specifically, no confidence in the impact of error reporting and no established culture of reporting were common barriers. This is concordant with other studies and resonates with the statement in one of them that “remaining silent about safety concerns is a common phenomenon in oncology” [31,32]. In contrast to many healthcare systems, the aviation industry has successfully improved safety over the past number of decades through the adoption of a blame-free culture and encouragement of reporting without fear of reprisal [33]. As outlined in ‘Europe's Beating Cancer Plan’, the health sector lags in exploiting the potential of relevant data [26]. The Institute of Medicine's publication ‘Delivering High-Quality Cancer Care: Charting a New Course for a System in Crisis’ presents a conceptual framework for improving cancer care quality and emphasises the importance of gathering and translating safety data into clinical practice, quality measurement and performance improvement [28].

This study did not include fear of litigation as a barrier respondents could choose. However, it is a common barrier particularly in radiology as there can be a reluctance to report discrepancies due to a lack of legal protection [34]. In 2009, the Faculty of Radiologists in Ireland initiated a national quality improvement programme which was the first of its kind worldwide. The programme's guidelines address communication of significant findings, peer review and reporting of discrepancies [34]. The European and American professional bodies for radiation oncology both have quality frameworks. Although each speciality may have internal quality systems, an overarching quality framework which tracks the patient from entry into the system to exit, that is their trajectory, may be lacking.

However, medical error reduction is vital because the impacts can be significant as demonstrated in this study. Most patients reported that their experience had physical and emotional impacts on them which were often long-term or permanent. Almost 90% of doctors reported being impacted emotionally however, it was more likely to be short-term. Medical error impacts also extend to doctors’ career choices with 25% considering early retirement because of their experiences. This increased to almost 50% for those involved in a negligence claim. The workforce implications of these findings are concerning particularly because a shortage of 10 million healthcare workers is predicted by 2030 and cancer rates are expected to increase by 45% [28,35]. A dilemma becomes apparent when medical error experiences may lead to reduced staff but most doctors in this study are already concerned about the impact staff shortages, high workloads and burn-out have on medical error. The potential loss of experienced doctors to any healthcare system is hugely concerning and the safety risks posed by inadequate staffing are well known but, as mentioned, may worsen in the next decade [36].

Communication errors were the type of adverse event that concerned the highest proportion of doctors. This demonstrates a recognition that communication is often implicated in oncological adverse events and can be a reason for litigation however, legal action seldom addresses this [30,37,38]. One potential solution is open disclosure. Its significance in oncology grew after a controversy involving Ireland's cervical cancer screening programme, CervicalCheck, which resulted in 360 legal claims [13]. A scoping inquiry found that CervicalCheck was operating at international standards but reported that Ireland's “practice in relation to open disclosure is deeply contradictory and unsatisfactory” [13]. This has been addressed by the enactment of the Patient Safety (Notifiable Incidents and Open Disclosure) Act 2023 which makes open disclosure mandatory for a list of adverse events [39]. Communication has long been recognised as a core skill for cancer clinicians and yet it continues to be a source of concern in this study.

Aside from communication, doctors were more concerned about administrative errors and poorly organised systems for record-keeping, appointments and follow-up than patients. While these issues can be multifaceted, one step to address them would be the implementation of an electronic health record [26]. As outlined in ‘Europe's Beating Cancer Plan’, “electronic health records are set to become crucial tools in cancer prevention and care” [26]. An integrated, standardised system would also facilitate Europe's goal of “making the most of data and digitalisation in cancer prevention and care” [26]. The importance of appropriate cyber security during all digital development became apparent during the 2021 cyber-attack on the Irish healthcare service [40].

Patient safety improvement and medical error reduction are not new concepts. However, this study demonstrates that breast cancer patients and doctors have patient safety concerns and experience of medical error. This study's strengths include patient insights related to contemporary practice and inputs from multiple disciplines many of whom had extensive clinical experience. The retrospective nature of this study made it sensitive to selection bias. This study is also subject to the limitations of much patient safety research including reliance on self-reported events, outcome and hindsight bias, lack of data on harm related to the event or whether the error would have altered management.

## 5 Conclusion

A significant prevalence of medical error and its consequent personal and professional impacts on breast cancer care in Ireland was noted in this study. Over half of doctors and a quarter of patients felt medical error prevention measures were inadequate. A greater proportion of doctors were concerned about adverse events and causative factors than patients. Underreporting was common and reasons cited included unclear reporting structures and a lack of confidence in the difference it would make. Multifaceted impacts of medical error on physical and emotional health were noted in both groups and also influenced doctors’ career choices. Integrating lessons from these findings could improve breast cancer care in Ireland.

## Declaration of interest statement

SOR has received income from the State Claims Agency and Medical Protection Society for expert reports related to litigation claims.

## CRediT authorship contribution statement

**Clara Forrest:** Conceptualization, Data curation, Formal analysis, Funding acquisition, Investigation, Methodology, Project administration, Resources, Software, Writing – original draft, Writing – review & editing. **Martin J. O'Sullivan:** Writing – original draft, Writing – review & editing. **Max Ryan:** Writing – original draft, Writing – review & editing. **Colm O'Tuathaigh:** Writing – original draft, Writing – review & editing. **Tara Jane Browne:** Writing – original draft, Writing – review & editing. **Kathy Rock:** Writing – original draft, Writing – review & editing. **Mary Jane O'Leary:** Writing – review & editing. **Deirdre Madden:** Conceptualization, Writing – original draft, Writing – review & editing. **Seamus O'Reilly:** Conceptualization, Writing – original draft, Writing – review & editing.
