# Supplementary material for: Patients' and doctors’ views and experiences of the patient safety trajectory of breast cancer care
Source: Breast. 2024 Feb 29;75:103699. doi: 10.1016/j.breast.2024.103699 (PMC10943021; doi:10.1016/j.breast.2024.103699)
Supplement: Multimedia component 1 [file mmc1.docx]

**Supplementary Material**

Table 1S: Demographics of Patients' and Doctors' Contrasting Experiences of Medical Error During Breast Cancer Care

| *Table 2: Demographics of patients* | | *Demographics of doctors* | |
| --- | --- | --- | --- |
| Number | 30 | Number | 48 |
| Gender (%) |  | Gender (%) |  |
| Female | 30 (100.0) | Female | 26 (54.2) |
| Male | 0 | Male | 21 (43.8) |
| Age groups (%) |  | Prefer not to say | 1 (2.1) |
| 18-34 | 3 (10.0) | Age groups (%) |  |
| 35-39 | 2 (6.7) | 25-34 | 5 (10.4) |
| 40-44 | 4 (13.3) | 35-39 | 3 (6.3) |
| 45-49 | 3 (10.0) | 40-44 | 8 (16.7) |
| 50-54 | 1 (3.3) | 45-49 | 8 (16.7) |
| 55-59 | 6 (20.0) | 50-54 | 9 (18.8) |
| 60+ | 11 (36.7) | 55-59 | 4 (8.3) |
| Patient or family member work in healthcare (%) | | 60+ | 11 (22.9) |
| Yes | 10 (33.3) | Job title (%) |  |
| No | 20 (66.7) | Consultant | 43 (89.6) |
| Residence (%) |  | Senior/ specialist registrar | 5 (10.4) |
| Rural area | 8 (26.7) | Junior registrar | 0 |
| Small town | 12 (40.0) | Field of medicine |  |
| Suburb | 8 (26.7) | Medical oncology | 13 (27.1) |
| City | 2 (6.7) | Radiation oncology | 10 (20.8) |
| Other | 0 | Radiology | 12 (25.0) |
| Currently have private health insurance (%) | | Surgery | 8 (16.7) |
| Yes | 17 (56.7) | Palliative medicine | 1 (2.1) |
| No | 13 (43.3) | Pathology | 4 (8.3) |
| Other | 0 | Experience with breast cancer patients (%) | |
| First diagnosed with breast cancer (%) | | < 1 year | 0 |
| < 1 year | 5 (16.7) | 1-2 years | 3 (6.3) |
| 1-2 years | 5 (16.7) | 3-5 years | 2 (4.2) |
| 3-4 years | 6 (20.0) | 5-10 years | 5 (10.4) |
| 5-6 years | 3 (10.0) | 10-20 years | 18 (37.5) |
| 7-8 years | 2 (6.7) | > 20 years | 20 (41.7) |
| 9-10 years | 1 (3.3) | Practice in a private capacity (%) |  |
| > 10 years | 8 (26.7) | Yes | 24 (50.0) |
| Unsure | 0 | No | 24 (50.0) |
| Error result in a medical negligence claim (%) |  | Error result in a medical negligence claim (%) | |
| Yes | 4 (13.3) | Yes | 13 (27.1) |
| Breast cancer type (%) |  |  |  |
| Hormone sensitive invasive ductal carcinoma | 4 (13.3) |  |  |
| Ductal carcinoma in-situ | 1 (3.3) |  |  |
| HER2 positive carcinoma | 2 (6.7) |  |  |
| Triple negative carcinoma | 6 (20.0) |  |  |
| Invasive lobular carcinoma | 1 (3.3) |  |  |
| Metastatic carcinoma | 7 (23.3) |  |  |
| Unsure | 7 (23.3) |  |  |
| Other | 2 (6.7) |  |  |

Table 2S: Demographics of Patients' and Doctors' Contrasting Patient Safety Concerns During Breast Cancer Care

| *Table 5: Demographics of patients* | | *Demographics of doctors* | |
| --- | --- | --- | --- |
| Number | 154 | Number | 44 |
| Gender (%) |  | Gender (%) |  |
| Female | 150 (97.4) | Female | 31 (70.5) |
| Male | 4 (2.6) | Male | 12 (27.3) |
| Age groups (%) |  | Prefer not to say | 1 (2.3) |
| 18-34 | 2 (1.2) | Age groups (%) |  |
| 35-39 | 8 (5.2) | 25-34 | 10 (22.7) |
| 40-44 | 18 (11.7) | 35-39 | 5 (11.4) |
| 45-49 | 16 (10.4) | 40-44 | 9 (20.5) |
| 50-54 | 24 (15.6) | 45-49 | 8 (18.2) |
| 55-59 | 31 (20.1) | 50-54 | 4 (9.1) |
| 60+ | 55 (35.7) | 55-59 | 6 (13.6) |
| Patient or family member work in healthcare (%) | | 60+ | 2 (4.5) |
| Yes | 32 (20.8) | Job title (%) |  |
| No | 122 (79.2) | Consultant | 31 (70.5) |
| Residence (%) |  | Senior/ specialist registrar | 12 (27.3) |
| Rural area | 52 (33.8) | Junior registrar | 1 (2.3) |
| Small town | 41 (26.6) | Field of medicine |  |
| Suburb | 31 (20.1) | Medical oncology | 14 (31.8) |
| City | 28 (18.2) | Radiation oncology | 11 (25.0) |
| Other | 2 (1.3) | Radiology | 7 (15.9) |
| Currently have private health insurance (%) | | Surgery | 8 (18.2) |
| Yes | 86 (55.8) | Palliative medicine | 4 (9.1) |
| No | 65 (42.2) | Pathology | 0 (0) |
| Other | 3 (1.6) | Experience with breast cancer patients (%) | |
| First diagnosed with breast cancer (%) | | < 1 year | 3 (6.8) |
| < 1 year | 31 (20.1) | 1-2 years | 1 (2.3) |
| 1-2 years | 26 (16.9) | 3-5 years | 7 (15.9) |
| 3-4 years | 45 (29.2) | 5-10 years | 7 (15.9) |
| 5-6 years | 14 (9.1) | 10-20 years | 15 (34.1) |
| 7-8 years | 6 (3.9) | > 20 years | 11 (25.0) |
| 9-10 years | 6 (3.9) | Practice in a private capacity (%) |  |
| > 10 years | 25 (16.2) | Yes | 18 (40.9) |
| Unsure | 1 (0.6) | No | 26 (59.1) |
| Breast cancer type (%) |  |  |  |
| Hormone sensitive invasive ductal carcinoma | 35 (22.7) |  |  |
| Ductal carcinoma in-situ | 10 (6.5) |  |  |
| HER2 positive carcinoma | 27 (17.5) |  |  |
| Triple negative carcinoma | 10 (6.5) |  |  |
| Invasive lobular carcinoma | 6 (3.9) |  |  |
| Metastatic carcinoma | 22 (14.3) |  |  |
| Unsure | 39 (25.3) |  |  |
| Other | 5 (3.1) |  |  |
